# Genome assembly of a symbiotic balantidia (*Balantidium ctenopharyngodoni*) in fish hindgut

**DOI:** 10.1038/s41597-024-03142-1

**Published:** 2024-03-28

**Authors:** Weishan Zhao, Jie Xiong, Ming Li, Xialian Bu, Chuanqi Jiang, Guangying Wang, Jing Zhang, Wenxiang Li, Hong Zou, Wei Miao, Kai Chen, Guitang Wang

**Affiliations:** 1 Institute of Hydrobiology, Chinese Academy of Sciences, Wuhan, 430072 China; 2 Protist 10,000 Genomics Project (P10K) Consortium, Wuhan, China; 3 Key Laboratory of Breeding Biotechnology and Sustainable Aquaculture, Institute of Hydrobiology, Chinese Academy of Sciences, Wuhan, 430072 China

**Keywords:** Comparative genomics, Parasite genomics

## Abstract

*Balantidium ctenopharyngodoni* is identified as the sole ciliate species that exclusively resides within the hindgut of grass carp with high prevalence and intensity. In this study, the successful cultivation of *B. ctenopharyngodoni* enabled us to collect enough cells for genome sequencing. Consequently, we acquired a high-quality genome assembly spanning 68.66 Mb, encompassing a total of 22,334 nanochromosomes. Furthermore, we predicted 29,348 protein-coding genes, and 95.5% of them was supported by the RNA-seq data. The trend of GC content in the subtelomeric regions of single-gene chromosomes was similar to other ciliates containing nanochromosomes. A large number of genes encoding carbohydrate-binding modules with affinities for starch and peptidoglycans was identified. The identification of mitochondrion-related organelles (MROs) within genome indicates its well-suited adaptation to the anaerobic conditions in the hindgut environment. In summary, our results will offer resources for understanding the genetic basis and molecular adaptations of balantidia to hindgut of herbivorous fish.

## Background & Summary

Ciliates are a diverse group of protozoa, characterized by the presence of both somatic macronucleus and germ-line micronucleus within a single cell. Most ciliates are free-living, but some are commensals or parasites of other organisms^1–3^. *Balantidium ctenopharyngodoni* is an obligate intestinal ciliate and possibly an opportunistic pathogen of grass carp^4,5^. It possesses a spindle-like, highly elastic, and thick body, which enables it to navigate through the gaps between mucosal folds in the hindgut^4^. This particular ciliate species is notably the sole ciliate parasite discovered in the digestive tract of grass carp older than one year^4,6^. This presence suggests that *B. ctenopharyngodoni* is closely associated with the digestion and utilization of plant food by grass carp^6^. Furthermore, *B. ctenopharyngodoni* showcases a distinctive tissue-specificity, being primarily found in the hindgut of grass carp, particularly within the segment spanning 6–10 cm before the anal opening^4^. Thus, it might have developed unique capabilities to cope with selective pressures of anaerobic niches, and this might make it a good model for studying adaptive evolution and the relationships between intestinal ciliates and hosts.

High-quality genome data plays a crucial role in comprehending how *B. ctenopharyngodoni* thrives in the anaerobic environment of the hindgut in herbivorous fish, as well as elucidating the sources of its energy. Indeed, obtaining a high-quality genome of this ciliate is constrained by two main challenges: (1) the species cannot be cultured extensively *in vitro* on a large scale; and (2) the existence of prokaryotic endosymbiotic bacteria poses difficulties in obtaining a pure genome. Most ciliates, especially the species inhabiting the digestive tract and living in extreme conditions, are very hard to culture. Thus, only a few ciliate species (less than 1%) have been sequenced at the genome level, most of which are free-living (e.g. species belonging to Oligohymenophorea and Spirotrichea)^7–10^.

In a previous study, we successfully developed an anaerobic culture method for *B. ctenopharyngodoni* using the BCM medium (an artificial medium for *in vitro* cultivation of *B. ctenopharyngodoni*)^6,11^, which allowed us to obtain a sufficient number of cells for genome sequencing. It was the first *in vitro* cultivation medium successfully developed for the growth of an intestinal ciliate from freshwater fish. Furthermore, we have established a systematic analytical process for decontaminating ciliate genome data, resulting in a pure genome acquired through this strategy. Additionally, we conducted gene prediction and functional annotation of *B. ctenopharyngodoni*. Finally, we identified genes and pathways associated with carbohydrate metabolism and energy metabolism. These will serve as valuable genetic resources for elucidating the adaption of *B. ctenopharyngodoni* to anaerobic hindgut of its host, and for further applying to grass carp aquaculture.

## Methods

### Sample culture and collection

**Fig. 1:**
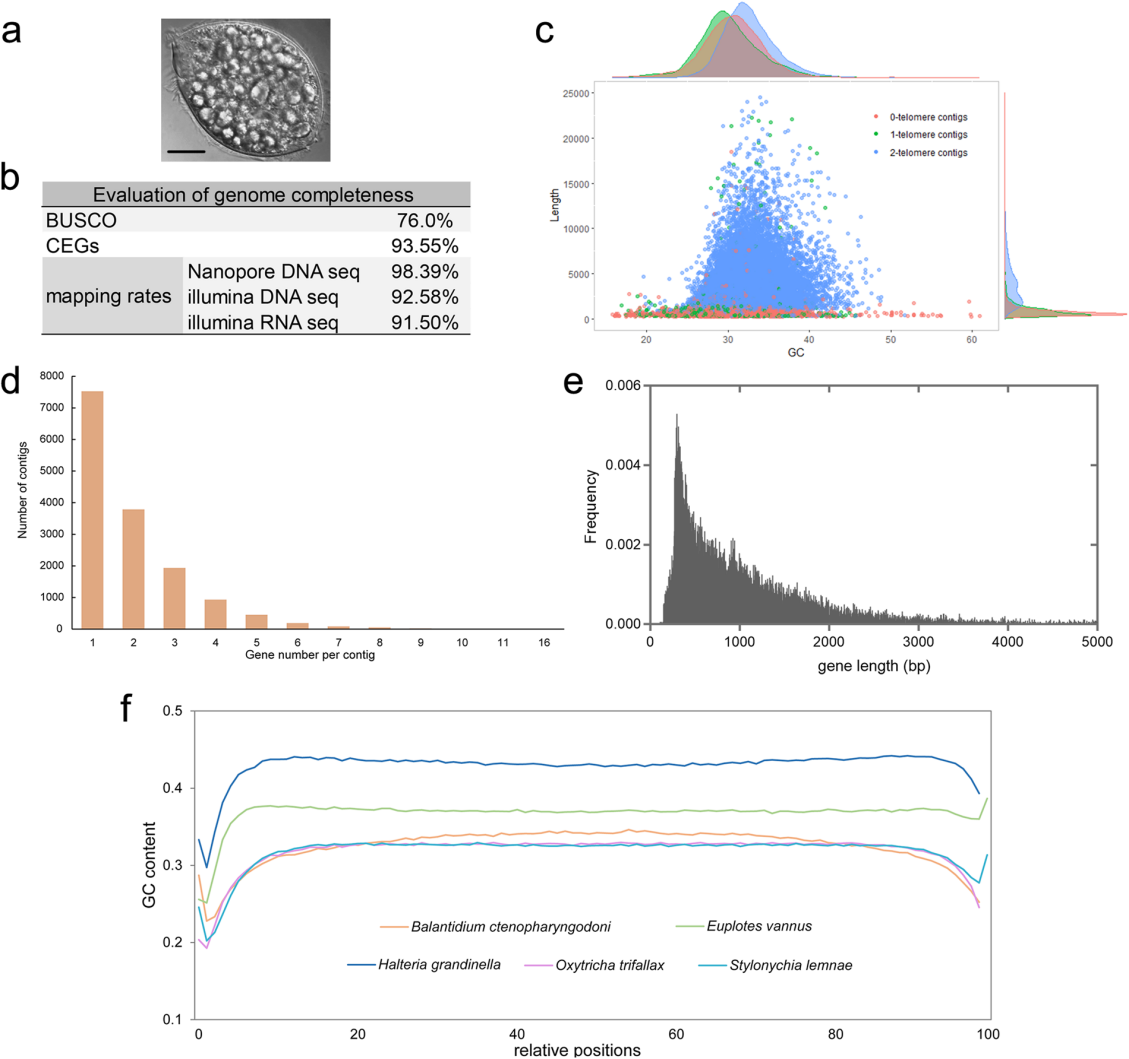
Overviews and characteristics of cell, genome, and gene in *Balantidium ctenopharyngodoni*. (**a**) living specimen of cultured *B. ctenopharyngodoni*, showing the starch granules in the cell. Scale bar = 20 μm. (**b**) Completeness evaluation of genome assembly. (**c**) GC content and length distribution of all, 2-telomere, 1-telomere, and 0-telomere contigs. (**d**) Statistics on gene numbers of contigs. (**e**) Length distribution of predicted genes. (**f**) Sliding-window analysis of GC content of single-gene chromosomes in five ciliates. Window size was 1% of the single-gene chromosomes.

*Balantidium ctenopharyngodoni* were initially isolated from the hindgut of grass carp captured from Liangzi Lake, Hubei province, China. They were maintained in the BCM medium in our lab, including regular transfers to a fresh BCM medium, as described before^6,12^. We isolated a single balantidia cell from the culture using a pulled glass pipette and used it to build a single-cell clone. All cells collected for sequencing in this study were derived from this single clone (Fig. 1a). For DNA samples, cells were harvested by successive daily sampling with a pipette, washed with sterile 0.65% saline solution three times to reduce bacterial contamination, and lysed with a urea buffer (20 mM Tris-HCl, 0.7 M NaCl, 20 mM EDTA, 2% SDS, 42% Urea). For RNA samples, cells were stabilized in RNAprotect Cell Reagent (Qiagen, USA), and stored at −80 °C.

### DNA and RNA sample preparation and sequencing

#### Total DNA and RNA extraction

Total DNA was extracted using a phenol-chloroform-isoamyl alcohol DNA extraction method (25:24:1), then the supernatant was transferred to a new microcentrifuge tube and re-extracted using a mixture of chloroform: isoamyl alcohol (24:1). The supernatant was precipitated with sodium acetate after being centrifuged at 12,000 rpm for 5 min. The DNA pellet was washed with 70% cold ethanol and resuspended in double distilled water.

Total RNA was extracted using RNeasy Protect Cell Mini Kit (Qiagen, USA) according to the manufacturer’s protocol.

#### Nanopore sequencing

Approximately 8 μg of DNA was used to construct sequencing libraries using the 1D Ligation sequencing kit SQK-LSK108 according to the manufacturer’s instructions (Oxford Nanopore Technologies, UK). The prepared libraries were loaded onto R9.4 FlowCells and sequenced using the PromethION sequencer (Oxford Nanopore Technologies, UK) at the Genome Center of Nextomics (Wuhan, China). The collected fast5 files were basecalled using the Guppy v1.8 software, and high-quality reads were used for downstream analysis.

#### Illumina sequencing

The DNA sequencing library was built followed by a series of treatments, such as terminal repairing, adaptor adding and PCR processing. For transcriptome sequencing, the library was generated using NEBNext®Ultra™ RNA Library Prep Kit following the manufacturer’s recommendations. The library quality was assessed on the Agilent Bioanalyzer 2100 system (Agilent Technologies, USA).

DNA and RNA libraries were sequenced with paired-end reads on Illumina NovaSeq. 6000 sequencing platform (Illumina, USA).

#### Genome assembly

With two different sequencing strategies, we obtained 18.8 Gb of long reads and 16.5 Gb of short reads. The long reads were used as the genome skeleton, and the short reads were used to correct the primary assembled genome. A hybrid assembly method was conducted to obtain a high-quality *B. ctenopharyngodoni* genome assembly.

The process were as follows: (1) Clean data were obtained by removing reads containing adapters and low-quality reads from raw data of paired-end reads using the FASTX-Toolkit, and then assembled using MEGAHIT v1.2.9^13^. Putative telomeric repeats of (CCCCAAT)n were identified in the assembly using the TRAP tool in ScaMPI^14^, and verified manually. NECAT was used to correct high-quality Nanopore sequencing reads^15^. Via the verification by PCR amplification, we found that some assembled contigs (~3%) and corrected Nanopore reads (~48%) had embedded telomeric sequences that were chimeras or sequencing artifacts of chromosomes (Fig. S1a,b, Table S1). The embedded telomeric sequences in the assembly and Nanopore reads were cut off and the three repeats of the telomeric sequence were added manually. (2) The treated Nanopore reads capped with at least one telomere sequence were selected to cluster with the assembled contigs, and the redundant sequences were removed with CD-HIT version 4.8.1 (-c 0.95)^16^. (3) The potential contaminants were identified and removed using iGDP^17^ and by searching against the NCBI NR database using Blastx program. (4) Genome polishing was performed twice using Pilon version 1.23 based on the paired-end reads^18^.

The final assembly of the *B. ctenopharyngodoni* genome is 68.66 Mb, containing 22,334 contigs with a mean GC content of 32.78%. Among these, 15,537 contigs (62.23 Mb, 90.6%) of the final assembly were capped with telomeres at both ends. Additionally, 1,732 contigs (2.21 Mb) contained a single telomere, indicating we have acquired a high-quality genome, while 5,065 contigs (4.22 Mb) did not contain any telomeric sequences (Table 1 and Table S2). The genome exhibits high-quality based on the genome evaluation (Fig. 1b). Notably, we have successfully assembled the complex rDNA sequences into independent chromosomes, a feature unique to ciliate genetics compared to other organisms. The GC content of all contigs was nearly the same as the GC content of 2-telomere contigs, which were considered to represent fully assembled chromosomes (Table S2, Fig. 1c). Although the number of 0-telomere contigs accounted for ~23% of the draft genome, their size was relatively small (only ~6% of the total size) with a mean length of 834 bp (Table S2). Almost all contigs had lengths of less than 25 kb, and ~26% of contigs were less than 1 kb. The mean length of all contigs was 3,074 bp (Table S2). This indicated that the macronuclear genome of *B. ctenopharyngodoni* was composed of extremely fragmented chromosomes, similar to some ciliates in the class Spirotrichea (Table 1).

**Table 1:** Comparison of genome characteristics in seven ciliates.

	*Balantidium ctenopharyngodoni*	*Entodinium caudatum*	*Oxytricha trifallax*	*Stylonychia lemnae*	*Halteria grandinella*	*Euplotes vannus*	*Tetrahymena thermophila*
Genome size (Mb)	68.66	92.08	67.16	50.16	64.05	85.09	103.35
Number of contigs	22334	30632	22363	19840	40422	38245	181
GC content (%)	32.78	21.5	31.4	31.7	43.1	36.9	22.3
N50 (bp)	4603	4075	3736	3290	2066	2685	929.7 kb
Number of 2-telomere contigs	15537	11371	13918	15908	16459	25507	181
2-telomere contigs size (Mb)	62.23	31.60	44.13	43.88	34.58	60.21	103.35
Mean 2-telomere contig length (bp)	4005	2779	3171	2759	2101	2361	571.0 kb
Size of 2-telomere contigs/total genome size (%)	90.6	34.3	65.7	87.5	54.0	70.8	100
Gene number	29348	—*	24578	20740	17815	43040	26258
Mean gene length (bp)	1235	—	2088	1860	1107	1460	2453
Number (Proportion (%)) of gene on 2-telomere contigs	28141 (95.9)	—	16322 (66.4)	18394 (88.7)	13114 (73.6)	33615 (78.1)	26258 (100)

*Data are unavailable in public database.

### Gene prediction and functional annotation

#### Protein coding gene prediction and features

RNA-seq data were trimmed and filtered by FASTQ Quality Trimmer/Filter in the FASTX-Toolkit (http://hannonlab.cshl.edu/fastx_toolkit/), then clean data were *de novo* assembled and referenced-guided assembled using Trinity v2.1.1^19^ and Tophat v2.1.0 - Cufflinks v2.2.1^20,21^ pipeline with default settings, respectively. Then three methods were used to predict gene models: ab initio gene prediction, RNA-seq-based prediction and homology-based prediction. For the ab initio gene prediction, the complete transcripts were filtered to train the gene prediction software programs Augustus v3.3.3^22^ and GlimmerHMM v3.0.4^23^, and the generated training parameters were used to ab initio predict gene models. For the RNA-seq-based prediction, gene models were predicted by the PASA pipeline^24^, then AATpackage r03052011^25^ was used to annotate genes based on cDNA evidence. For homology-based prediction, protein sequences of *Euplotes crassus*, *Paramecium tetraurelia* and *Oxytricha trifallax* were downloaded from the Uniprot database^26^, and *Tetrahymena thermophile* proteins were retrieved from the Tetrahymena Genome Database (http://ciliate.org/index.php/home/welcome). Then, Scipio v1.4^27^ and AATpackage were used to predict protein homologies of *B. ctenopharyngodoni* based on the above protein database. Finally, all predicted gene models were merged by Evidence Modeler r2012-06-25 to generate a final integrated set of gene models^28^.

Consequently, we predicted 29,348 genes within the genome. Among these, 28,028 genes (95.5%) were supported by the RNA-seq data, indicating their active expression. The average gene length measured 1,235 bp, contributing to a cumulative size of 36.24 Mb. We further identified 14,116 well-assembled chromosomes, collectively harboring 28,141 genes that averages to approximately two genes per chromosome (Table 1, Table S2). About 75% of contigs contained one or two genes (Fig. 1d). Among these, 6,807 well-assembled chromosomes contained only one gene. The single-gene chromosomes had low GC content in the subtelomeric regions in comparison to the coding regions, which is similar to other ciliates containing nanochromosomes (Fig. 1f).

#### Identification of complete rRNA gene

Ribosomal RNA (rRNA) genes were identified using RNAmmer version 1.2^29^ and further confirmed using BLAST. Three small subunits of ribosomal RNA (18s rRNA) genes of *B. ctenopharyngodon*i were retrieved from the GenBank database (MK204639, KU170970, GU480804). The complete 5.8s and 28s rRNA sequences (accession number AF223570.1) of *Spathidium amphoriforme* (Haptoria, Litostomatea) were used to precisely identify the boundaries of 5.8s and 28s rRNA genes of *B. ctenopharyngodoni*. A complete 18s-5.8s-28s rRNA gene located in the complete chromosome was identified (Fig. S2). The lengths of 18s rRNA, 5.8s rRNA and 28s rRNA were 1635 bp, 151 bp and 3176 bp, respectively. The 18s rRNA gene identified in the genome had more than 99% identity with three 18s sequences of *B. ctenopharyngodoni* downloaded from GenBank.

#### Identification of transporters in *B. ctenopharyngodoni*

Membrane transport systems are important cellular components for ciliate, which play vital roles on regulation of ciliate behaviours, communications, and substances exchange, etc^30,31^. We conducted the identification of membrane transport proteins through gblast3 analysis within the BioV suite, utilizing the Transporter Classification Database (TCDB, accessed on October 20, 2022^32,33^. This meticulous approach led to the successful identification of 2,454 genes within the *B. ctenopharyngodoni* genome that potentially encode transporters. Among these, 627 genes belonged to the Ankyrin Repeat Domain-containing (Ank) Superfamily (Table S3), the majority of which (606, 96.7%) were nuclear pore complex proteins (1.I.1). In terms of voltage-gated ion channel (VIC) superfamily, the count of genes in *B. ctenopharyngodoni* were comparatively lower than that observed in other free-living ciliates (Table S3). Further analysis involved comparing the gene counts specific for calcium, potassium, and sodium ions, along with those non-specific for cations within the VIC superfamily. The outcomes revealed that only six predicted genes were annotated as VICs with Ca^2+^ as a substrate. Notably, just one gene belonged to the VIC family exhibiting specificity for Ca^2+^ (1.A.1.11.14), while the remaining five genes were identified as part of the Ryanodine-Inositol 1,4,5-triphosphate Receptor Ca^2+^ Channel (RIR-CaC) family (Table S3). Furthermore, the study identified 13 mitochondrial carrier (MC) proteins in the *B. ctenopharyngodoni* genome (Table S3).

#### Functional annotation and comparative genome analysis

**Fig. 2:**
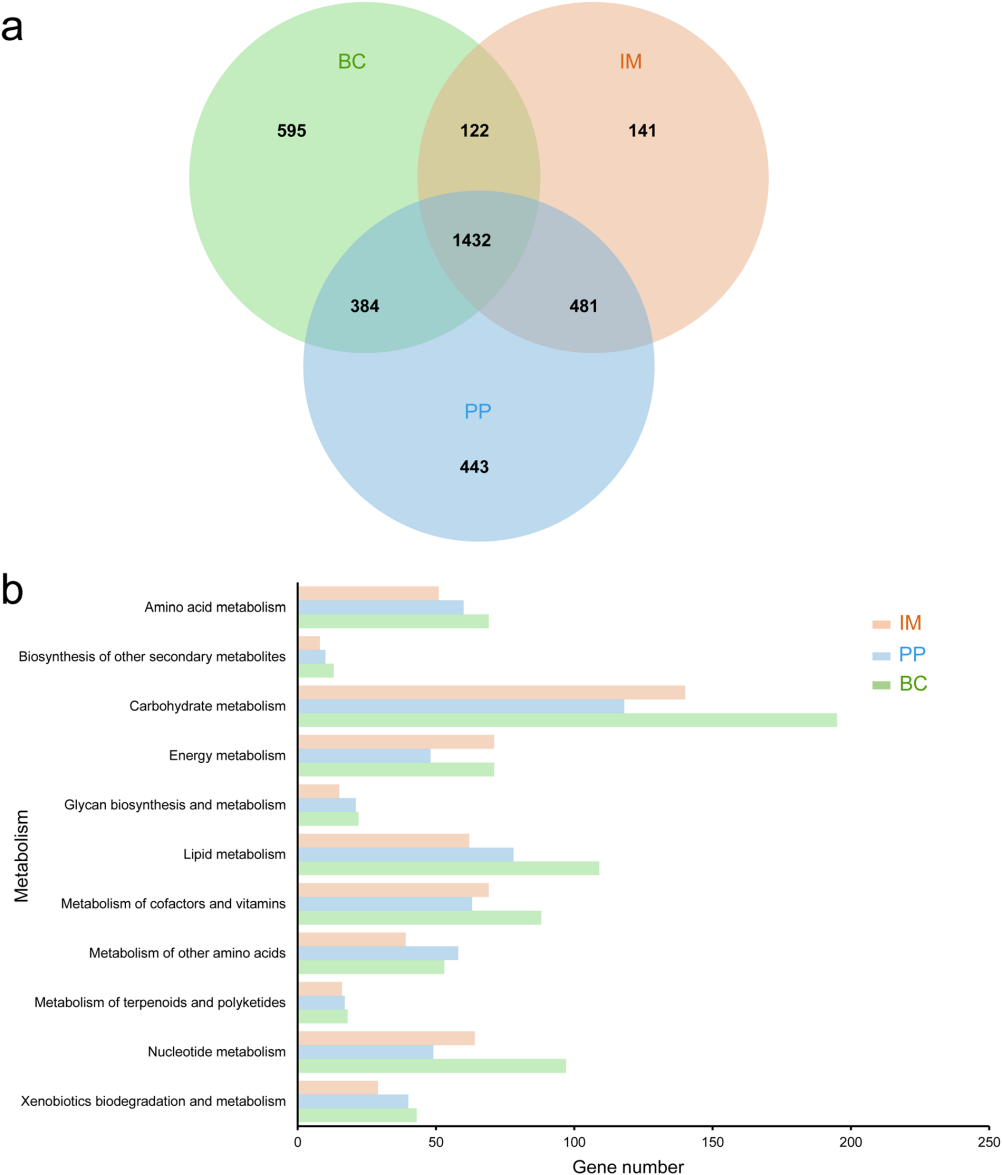
Comparison of function annotations in three fish ciliates. BC: *Balantidium ctenopharyngodoni*, IM: *Ichthyophthirius multifiliis*, PP: *Pseudocohnilembus persalinus*. M: metabolism, GIP: genetic information processing, EIP: environmental information processing, CP: cellular processes. (**a**) Number of KOs among *B. ctenopharyngodoni*, *I. multifiliis* and *P. persalinus*. (**b**) Statistics on genes of common KOs in metabolism pathways.

InterProScan version 5.52–86.0^34^ was used to annotate the functions of predicted protein sequences, and BLASTP searches were conducted against the non-redundant protein database (NR). The two results obtained above were subsequently imported into Blast2GO version 5.2.5^35^ to generate gene ontology annotations. All identified proteins were searched against the KAAS web server^36^, BlastKOALA server^37^ and KofamKOALA server^38^ to further gain KEGG annotations. The above results were integrated to produce more complete results. The enzyme commission number (EC number) was assigned according to the KO identifiers of proteins. A functional annotation was assigned to a total of 12,463 genes, out of which 6,709 predicted genes were specified by KO identifiers. Among 22 pathways, 17.0% of genes were involved in metabolism, 25.1% in genetic information processing, 22.3% in environmental information processing, and 35.6% in cellular processes (Fig. S3). Genes encoded within single-gene chromosomes were compared among five ciliates, in which 506 GO terms were commonly shared (Fig. S4a,b). The KEGG annotations of proteins in fish-related ciliates were also compared and analyzed. A total of 1432 KEGG orthologs (KOs) were shared by all three fish ciliates (Fig. 2a,b). *B. ctenopharyngodoni* exhibited 595 unique KOs, while *I. multifiliis* and *P. persalinus* had only 141 and 443 unique KOs, respectively (Fig. 2a). When considering shared KOs associated with metabolic pathways, *B. ctenopharyngodoni* displayed more genes linked to carbohydrate metabolism (Fig. 2b).

#### Annotation of carbohydrate-active enzyme genes

The annotation of carbohydrate-active enzymes (CAZymes) can be used to analyse the ability of an organism on assembling and breaking down the complex carbohydrates^39^. Besides, the infection of *B. ctenopharyngodoni* is closely related to herbivorous diet of grass carp. To identify these genes in *B. ctenopharyngodoni*, all predicted protein-coding genes were searched against the dbCAN2 CAZyme domain in the HMM database^40^ using hmmscan^41^, and in the CAZyme database^39^ using BLASTP. Both results were combined to generate the final CAZyme genes. Through the utilization of HMM and BLASTP methods, we successfully identified 228 carbohydrate-active enzymes, which included 16 families of 80 carbohydrate-binding modules (CBMs), 18 families of 60 glycoside hydrolases (GHs), 17 families of 57 glycoside transferases (GTs), 3 families of 4 polysaccharide lyases (PLs), 6 families of 24 carbohydrate esterases (CEs), and 2 families of 3 auxiliary activities (AAs). A total of 26 glycoside hydrolase family 13 (GH13) genes were identified, which accounted for a large proportion (43.33%) of all GH genes (Fig. S5a). Our results showed that genes encoding CBM20 with an affinity for starch and encoding CBM50 with an affinity for peptidoglycan had a proportion of 23.75% and 22.50%, respectively. Moreover, we identified 10 genes encoding CBM48, which can attach to GH13 module with glycogen-binding function (Fig. S5b). However, no cellulase gene was identified in the cellulose degradation pathway. As for the starch degradation pathway, our analysis identified 16 genes encoding amylases and glucosidases responsible for hydrolysis of starch and glycogen (Table S4). Additionally, pivotal enzymes involved in the conversion of glucose to amylopectin in the starch biosynthesis pathway, such as glgA and GBE1, were also discerned within the genome.

### Identification of Horizontal gene transfer (HGT) events

**Fig. 3:**
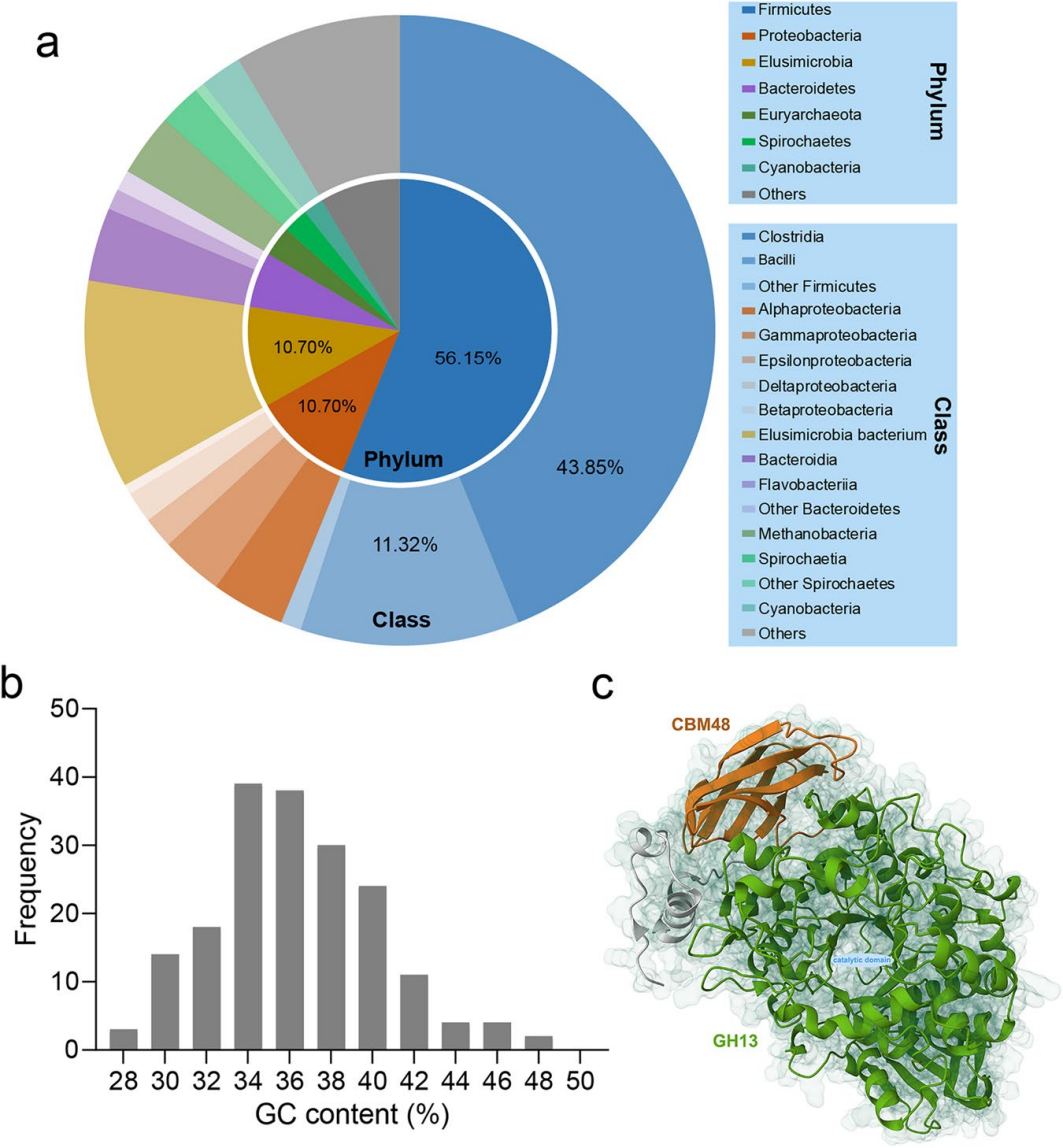
General information of HGT genes identified in *Balantidium ctenopharyngodoni* genome. (**a**) The distribution of bacterial donor species. (**b**) Length distribution of HGT genes. (**c**) Structure of type I pullulanase probably transferred from *Clostridium* sp., showing a TIM-barrel fold for catalytic domain.

All predicted genes were searched against the NCBI NR database with the E-value threshold of 1 × 10^−5^ according to Zhang, *et al*.^42^ and Xiong, *et al*.^10^. A total of 187 genes originating from prokaryotes were identified in *B. ctenopharyngodoni*^43^. Among these, the genes that are inferred to have been transferred from Firmicutes were the most prevalent, accounting for 105 genes. Within this category, Clostridia stood out as the primary source, contributing 82 genes to this gene transfer (Fig. 3a). All predicted HGT genes had a similar length distribution and frequencies of A, C, G, T at the third codon position to that of the total genome (Figs. 3b, S6).

A horizontally transferred type I pullulanase was selected to predict protein structure using AlphaFold2^44^, and then the best structure model was visualized and analyzed using Mol* Viewer^45^ (Fig. 3c). The identified pullulanase in *B. ctenopharyngodoni* has a binding domain of CBM48 and a TIM-barrel fold for the catalytic domain of GH13 (Fig. 3c), which hydrolyse 1,6-α-D-glucosidic linkages in pullulan or branched oligosaccharides to a long-linear α-D-glucan^46,47^.

### Prediction of enzymes in mitochondrion-related organelle

We formerly observed the mitochondrion-related organelles (MROs) in the cell of *B. ctenopharyngodoni* via transmission electron microscopy, no mitochondrial crista was found and the MRO shapes varied largely, from spherical to dumbbell-shaped. To investigate the MRO protein-coding genes in *B. ctenopharyngodoni*, the mitochondrial proteomes of humans and mice were retrieved from MitoCarta3.0 datasets^48^. The proteome of yeast was retrieved from the *Saccharomyces* Genome Database (https://www.yeastgenome.org/) and the mitochondrial proteins were extracted according to Sickmann, *et al*.^49^. The hydrogenosome proteins of *Trichomonas vaginalis* were extracted from all proteins retrieved from TrichDB (https://trichdb.org) according to Beltrán, *et al*.^50^. The mitochondrial proteins of *T. thermophila* were obtained from the supplementary Table 3 of Smith, *et al*.^51^. We also retrieved hydrogenase, pyruvate-formate lyase, succinyl-coa synthetase, and alternative oxidase from the UniProt database under the taxonomy of Intramacronucleate and Bacteria. Then we used the reciprocal best hits method in BLAST with an E-value of 1 × 10^−5^ to identify putative MRO protein orthologs. All identified MRO proteins were further annotated using KAAS^36^ and BlastKOALA^37^ servers.

**Fig. 4:**
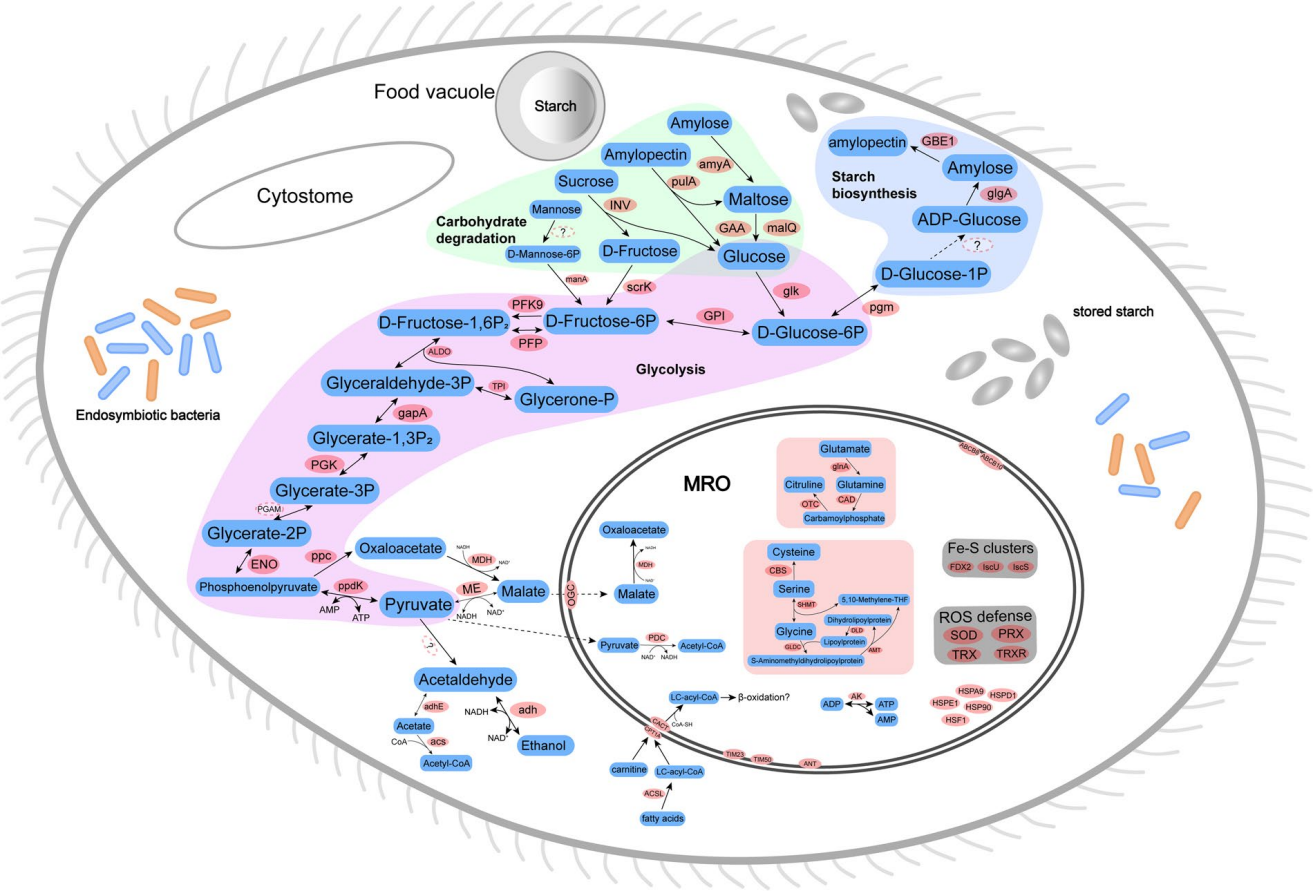
*In silico* reconstruction of major carbohydrate metabolism and other components in *Balantidium ctenopharyngodoni*, based on KEGG functional orthologs. Enzymes and proteins are indicated in pink oval, substrates are marked in blue, and undetected or undetermined enzymes in genome are marked as ‘?’ in dashed oval. Polysaccharide degradation pathways were in light green, glycogen biosynthesis was in light blue, glycolysis was in light purple, amino acid metabolisms were in light pink, ROS defense and Fe-S clusters were in grey. Abbreviations can be available in Figshare^43^.

We totally identified 159 genes encoding putative MRO proteins in *B. ctenopharyngodoni*. Among these, 98.1% (156 sequences) of putative MRO genes were annotated using BlastKOALA and KAAS servers^43^. Glycolysis is the backbone of carbon and energy metabolism^52^, so we identified homologs of enzymes involved in the glycolysis pathway (Fig. 4). For mitochondrial DNA, we searched the genome assembly and corrected Nanopore reads using BLAST, but no mitochondrial genome was found. Pyruvate:ferredoxin oxidoreductase (PFO) mediates the generation of acetyl-CoA in diverse anaerobic eukaryotes. We used the reciprocal best hit method to investigate the PFO in *B. ctenopharyngodoni*. No homologs of PFO were found, but a partial pyruvate dehydrogenase complex (PDC) was identified. Furthermore, We did not identify any genes belonging to the acetate:succinate CoA-transferase subfamily, nor succinyl-CoA synthetase (ASCT/SCS) proteins, it was reported that they were also not found in another vestibuliferid ciliate^53^. Enzymes involved in several amino acid metabolic pathways were detected; for example, we detected enzymes mediating the interconversion of cysteine, serine and glycine (Fig. 4).

Oxygen-scavenging enzymes, including superoxide dismutase (SOD), thioredoxin (TRX), thioredoxin reductase (TRXR) and peroxiredoxin (PRX), were identified in the MROs of *B. ctenopharyngodoni*. Twelve genes encoding superoxide dismutase (SOD) and peroxiredoxin (PRX) were identified in the genome of *B. ctenopharyngodoni* (six genes were predicted in MROs). All these genes were identified in telomere-capped chromosomes and supported by RNA-seq data (Table S5). In summary, MROs in *B. ctenopharyngodoni* is highly reduced, which lacks the TCA cycle, electron transport chain, mitochondrial genome, and cristae.

## Data Records

The genome assembly^54^ and raw sequencing data including Nanopore long reads (SRR26318080^55^) and Illumina short reads (SRR26318078^56^, SRR26318079^57^) have been submitted to the NCBI database under the BioProject accession number PRJNA1025258. Additionally, the sequencing data have also been deposited at National Genomics Data Center, Beijing Institute of Genomics, Chinese Academy of Sciences/China National Center for Bioinformation with Genome Sequence Archive (GSA) database accession number CRA011003^58^. Genome annotations, HGT genes, MRO proteins and list of abbreviations can be accessed through Figshare^43^.

## Technical Validation

We have developed a man-made medium exclusively suitable for *in vitro* cultivation of *B. ctenopharyngodoni* (BCM medium)^6,11^. Thus, the cell materials used for sequencing were a single-cell strain derived from one trophozoite of *B. ctenopharyngodoni* in the BCM medium.

Five criteria were adopted to assess the completeness of the assembled genome: (1) BUSCO analysis, (2) the mapping rates of the genomic Nanopore sequencing reads, (3) the mapping rates of Illumina DNA sequencing reads, (4) the mapping rates of Illumina RNA sequencing (RNA-seq) reads, and (5) the proportion of the core eukaryotic genes (CEGs). In detail, BUSCO analysis was conducted against the Alveolata lineages (–lineage_dataset alveolata_odb10)^59^. For the CEGs analysis, we used a two-step approach to identify them: first, the homologs were searched in the CEGs dataset (248 genes downloaded from http://korflab.ucdavis.edu/datasets/genome_completeness/index.html#SCT2) using BLASTP; then the Pfam-A HMM profiles of CEGs were searched using the E-value < 1e-3 to identify other CEGs that were not found in the last step. The proportion of CEGs was calculated after CEGs were identified in the genome of *B. ctenopharyngodoni*. The mapping rate of three types of sequencing reads onto the draft genome of *B. ctenopharyngodoni* was also calculated. For nanopore sequencing reads, the corrected reads were mapped onto the genome using Minimap2 version 2.22-r1101^60^. For paired-end reads and transcriptomic sequences, clean data were mapped onto the genome using Bowtie2 version 2.3.5.1^61^. All generated mapping results were used to calculate mapping rates of sequences using SAMtools v1.13^62^. The draft genome contained 76.0% of complete conserved orthologs within the Alveolata based on BUSCO analysis, and 232 of the 248 (93.55%) CEGs. The mapping rates of corrected Nanopore sequencing reads, Illumina DNA paired-end reads and RNA-seq were 98.39%, 92.58% and 91.50%, respectively (Fig. 1b).

### Supplementary information

Supplementary information

## Data Availability

The versions of the software employed in this study have been specified in the Methods section. The default parameter was used, if no parameter was provided. No custom code was used in this study for the curation and/or validation of the datasets.
