# Supplementary material for: Genome assembly of a symbiotic balantidia (Balantidium ctenopharyngodoni) in fish hindgut
Source: Sci Data. 2024 Mar 28;11:323. doi: 10.1038/s41597-024-03142-1 (PMC10978948; doi:10.1038/s41597-024-03142-1)
Supplement: Supplementary file 1 — Supplementary information [file 41597_2024_3142_MOESM1_ESM.pdf]

Supplementary Information

Table of Contents

Figure S1 ··· 1

Figure S2 ··· 2

Figure S3 ··· 3

Figure S4 ··· 4

Figure S5 ··· 5

Figure S6 ··· 6

Table S1 ··· 7

Table S2 ··· 8

Table S3 ··· 9

Table S4 ··· 10

Table S5 ··· 11

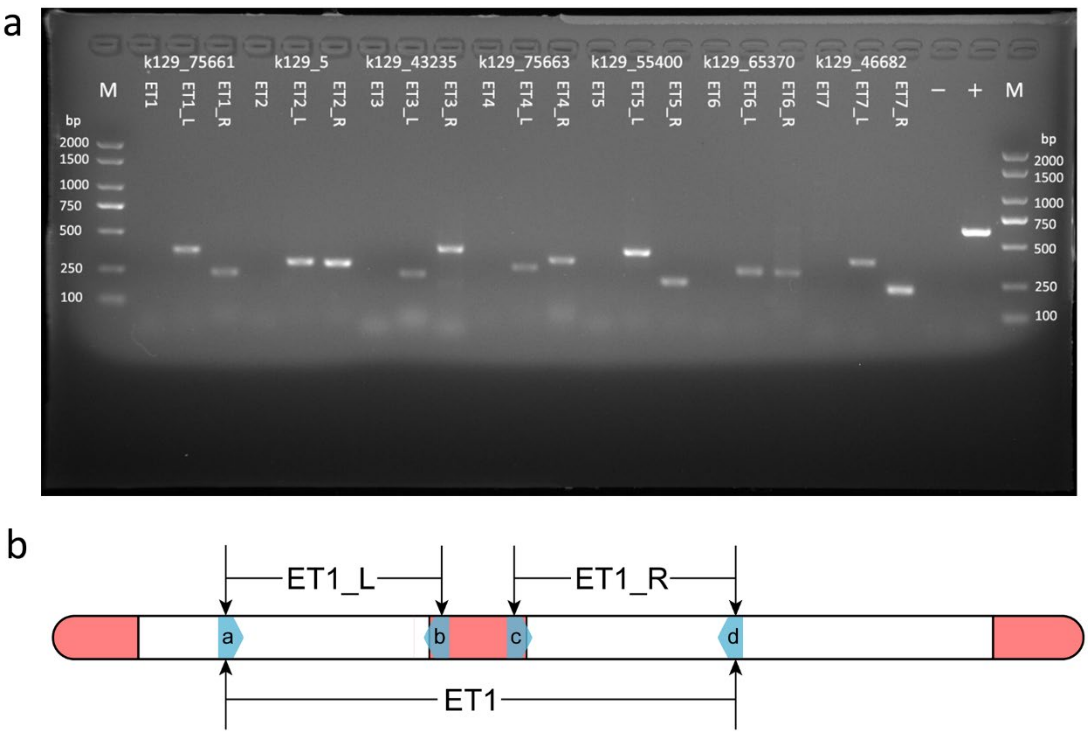

Figure S1. Verification of embedded telomere by PCR amplification. (**a**) Agarose gel electrophoresis of PCR products. M, DNA marker. −, negative control. +, positive control. (**b**) Example of amplified fragments and PCR primers. Telomeres were with color of brick red, a: forward primer of ET1 DNA fragment, b: reverse primer of ET1_L DNA fragment, c: forward primer of ET1_R DNA fragment, d: reverse primer of ET1 DNA fragment. Amplification regions were ET1, ET1_L, and ET1_R.

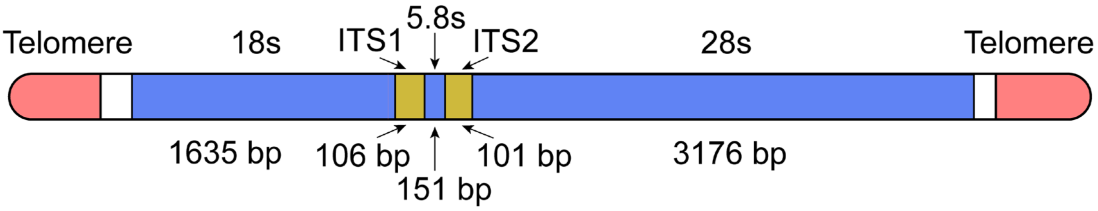

Figure S2. Illustration of complete ribosomal DNA identified in *Balantidium ctenopharyngodoni*.

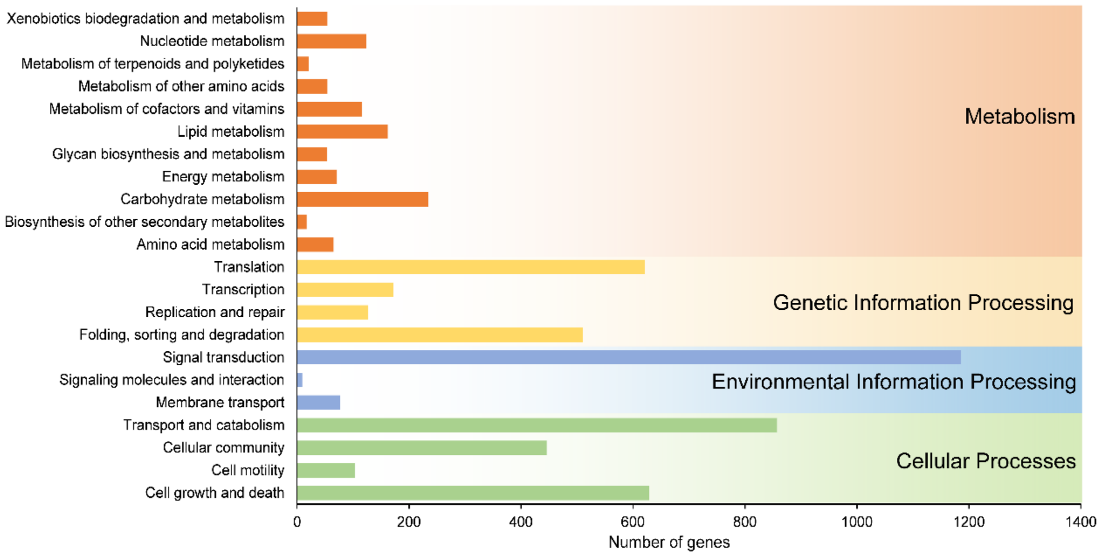

Figure S3. Functional annotation of predicted genes in *Balantidium ctenopharyngodoni* based on the KEGG pathways.

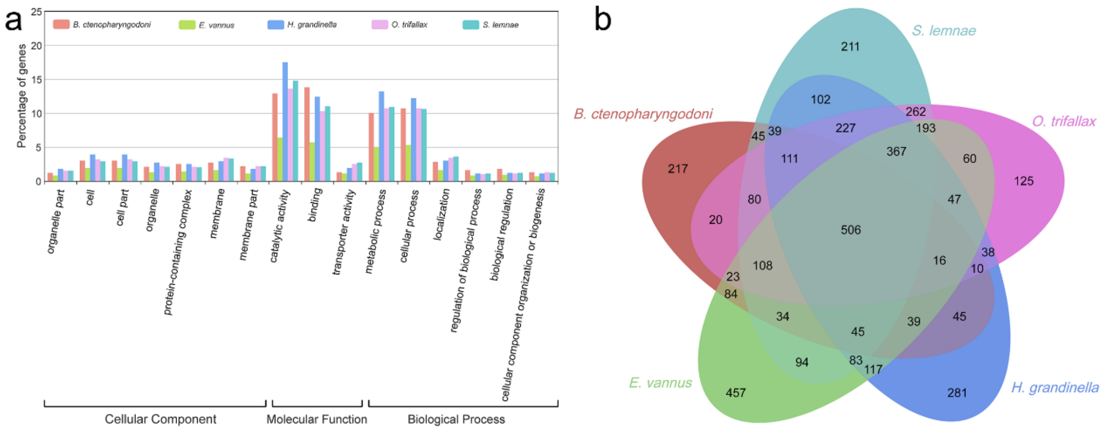

Figure S4. Functional comparison of chromosomes containing only one gene among the ciliates with nanochromosomes. (A) Enrichments of gene ontology. (B) Venn diagram of GO terms.

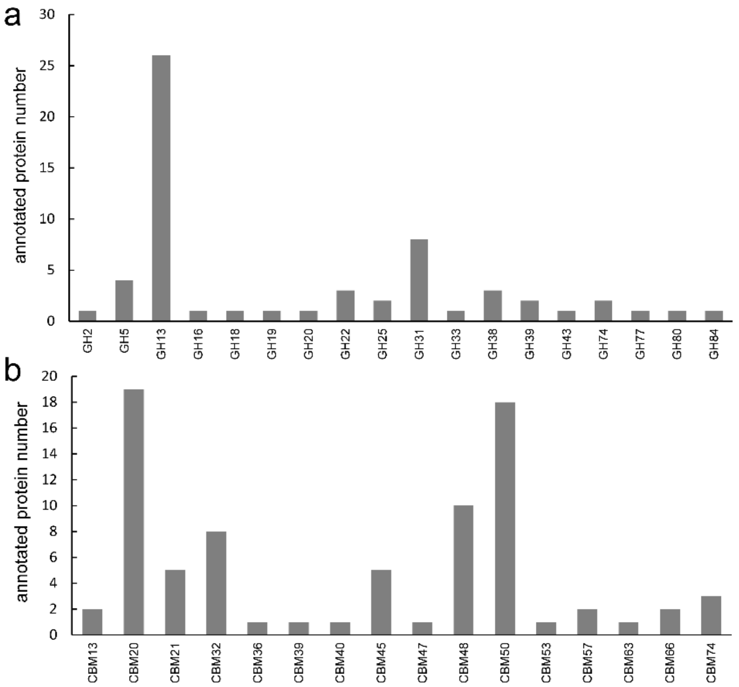

Figure S5. Distribution of carbohydrate-active enzymes in *Balantidium ctenopharyngodoni*. (**a**) Genes annotated as glycoside hydrolases (GH). (**b**) Genes annotated as carbohydrate-binding modules (CBM).

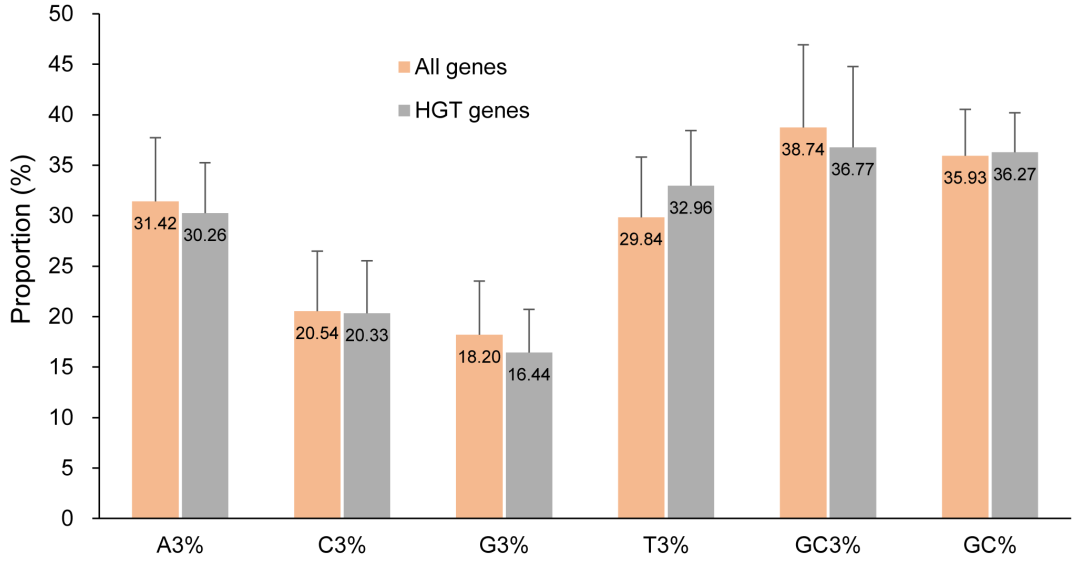

Figure S6. Comparison of nucleotide frequency between all protein-coding genes and HGT genes. A3%: The frequencies of adenine at the third codon positions. C3%: The frequencies of cytosine at the third codon positions. G3%: The frequencies of guanine at the third codon positions. T3%: The frequencies of thymine at the third codon positions. GC3%: The GC content in the third positions. GC%: The GC content of genes.

Table S1 Designed primers of verification of embedded-telomere contigs in pre-assembly genome

Pre-assembled contig ID	Amplified Fragment ID	Predicted Length	Forward Primer	Reverse Primer
	ET1	636	GTGATAACGGCAGAGTGAT	CAGAAGGTGGGTTGTGTAA
assem_75661	ET1_L	372	GTGATAACGGCAGAGTGAT	AATCCCCGTGTATCCTCC
	ET2_R	237	AATCCCCAACTGATTAGATG	CAGAAGGTGGGTTGTGTAA
	ET2	625	GCGATTTGAGGAATTTACGA	GAGCAATGAGAGGTGGATT
assem_5	ET2_L	298	GCGATTTGAGGAATTTACGA	AATCCCCAATACCCCACCT
	ET2_R	293	AATCCCCAAACTATGATTAG	GAGCAATGAGAGGTGGATT
	ET3	666	TGAGGGACTTACCGTGTT	AATGTCCGTGTTCGTGATA
assem_43235	ET3_L	248	TGAGGGACTTACCGTGTT	AATCCCCAGTTCACTCAAG
	ET3_R	398	AATCCCCAATCCCCATCC	AATGTCCGTGTTCGTGATA
	ET4	649	AGAGCAATGAAGACAAGGTA	GTATCTGATGATGTGAATGGG
assem_75663	ET4_L	289	AGAGCAATGAAGACAAGGTA	AATCCCCAATCTTTGAGTTC
	ET4_R	333	AATCCCCAATCCCCAATAACTAA	GTATCTGATGATGTGAATGGG
	ET5	689	AATGTCTTGTCTGGTAGTGT	GGGTAAATGCGTTGGTTAA
assem_55400	ET5_L	401	AATGTCTTGTCTGGTAGTGT	AATCCCCAATCCCCATTTCTT
	ET5_R	219	AATCCCCAAAGTGCTCTTAT	GGGTAAATGCGTTGGTTAA
	ET6	623	TACTGCTGATTGCTTGGTT	GAAGATGAAAGGAGAGATGAAG
assem_65370	ET6_L	286	TACTGCTGATTGCTTGGTT	AATCCCCAACCAATTCATAC
	ET6_R	289	AATCCCCAATCCCCATTATAT	GAAGATGAAAGGAGAGATGAAG
	ET7	617	TTGGAGAGTTCTTCGTCAC	GAGAAATGAGGAAAGGAAAGG
assem_46682	ET7_L	369	TTGGAGAGTTCTTCGTCAC	AATCCCCACTAATTTAGAAGC
	ET7_R	221	AATCCCCATCCATCGATTTTAC	GAGAAATGAGGAAAGGAAAGG

Table S2 Detailed information of the final assembly of *Balantidium ctenopharyngodoni*

Parameter	Total contigs	2-telomere contigs	1-telomere contigs	0-telomere contigs
Number of total contigs	22,334	15,537	1,732	5,065
Number of contigs >= 1 kb	16,525	14,908	602	1,015
Number of contigs >= 5 kb	4,181	4,090	53	38
Number of contigs >= 10 kb	643	602	30	11
Number of contigs >= 25 kb	4	2	2	0
Total length (bp)	68,661,084	62,231,406	2,207,402	4,222,276
Length of contigs >= 1 kb	65,132,217	61,765,227	1,561,251	1,805,739
Length of contigs >= 5 kb	32,282,248	31,310,441	651,388	320,419
Length of contigs >= 10 kb	8,448,240	7,801,138	501,137	145,965
Length of contigs >= 25 kb	114,518	57,934	56,584	0
Largest contig (bp)	31,440	28,985	31,440	18,515
Mean length (bp)	3,074	4,005	1,274	834
GC (%)	32.78	32.97	31.25	30.86

Table S3 Distribution of membrane transporters encoded in the genome of *Balantidium ctenopharyngodoni* and other ciliates

	Balantidium ctenopharyngodoni	*Ichthyophthirius multifiliis*	*Pseudocohnilembus persalinus*	*Tetrahymena thermophila*	*Paramecium tetraurelia*	*Oxytricha trifallax*	*Stylonychia lemnae*
TC superfamily							
Ank	627	254	978	1688	1270	2791	1094
PK	292	121	100	172	639	525	166
ArsA	94	38	51	177	120	150	76
VIC	84	71	79	385	429	235	104
MC	13	36	48	52	85	118	46
P-ATPase	67	45	27	78	99	107	43
MFS	107	60	61	161	163	326	188
APC	31	7	16	39	31	90	41
DMT	26	4	11	41	20	85	43
VIC							
calcium ion	6	20	9	30	77	19	13
potassium ion	35	42	62	330	304	125	56
sodium ion	2	0	2	3	3	6	3
non-specific cation	17	4	5	7	16	34	12

Table S4 Annotated proteins of *Balantidium ctenopharyngodoni* involved in starch degradation pathway

Enzyme	EC code	Gene id	CAZy code	RNA-seq coverage
Alpha-amylase	EC:3.2.1.1	BCP_12096.1	CBM21, GH13, GH13_32	100.00
		BCP_16156.1	CBM21, GH13, GH13_32	100.00
		BCP_1770.1	CBM21, GH13, GH13_32	100.00
		BCP_2051.1	GH13_1	100.00
		BCP_2624.2	GH13_1	100.00
		BCP_2853.8	CBM21, GH13, GH13_32	100.00
		BCP_3908.2	GH13_1	99.93
		BCP_7855.1	GH13, GH13_32	99.90
		BCP_9670.2	CBM21, GH13, GH13_32	100.00
Alpha-glucosidase	EC:3.2.1.20	BCP_12817.1	GH31	99.93
		BCP_1740.2	GH31	99.63
amylo-1,6-glucosidase	EC:3.2.1.33	BCP_186.2	CBM20, GH13_25	99.92
		BCP_3376.2	CBM48, GH13_25	98.05
		BCP_3376.3	CBM48	96.46
Pullulanase	EC:3.2.1.41	BCP_12468.1	CBM48, GH13_14	99.54
4-alpha-glucanotransferase	EC:2.4.1.25	BCP_4310.3	GH77	100.00

Table S5 Oxygen-scavenging enzymes of *Balantidium ctenopharyngodoni*

Enzyme	EC number	Protein id	Telomere-capped	Location	Transcriptome	mean depth
superoxide dismutase (SOD)	EC:1.15.1.1	BCP_2100.1	Yes		Mapped	772
		BCP_2100.2	Yes		Mapped	354
		BCP_699.1	Yes		Mapped	92
		BCP_7765.4	Yes	MRO	Mapped	2252
peroxiredoxin (PRX)	EC:1.11.1.24	BCP_12698.1	Yes	MRO	Mapped	3263
		BCP_16187.1	Yes		Mapped	741
		BCP_252.2	Yes	MRO	Mapped	245
		BCP_9060.1	Yes	MRO	Mapped	3207
thioredoxin (TRX)		BCP_12691.3	Yes		Mapped	216
		BCP_5365.1	Yes	MRO	Mapped	44
thioredoxin reductase (TRXR)	EC:1.8.1.9	BCP_12952.1	Yes		Mapped	66
		BCP_14573.1	Yes	MRO	Mapped	1252
